# Longitudinal homogenization of the microbiome between both occupants and the built environment in a cohort of United States Air Force Cadets

**DOI:** 10.1186/s40168-019-0686-6

**Published:** 2019-05-02

**Authors:** Anukriti Sharma, Miles Richardson, Lauren Cralle, Christopher E. Stamper, Juan P. Maestre, Kelly A. Stearns-Yoder, Teodor T. Postolache, Katherine L. Bates, Kerry A. Kinney, Lisa A. Brenner, Christopher A. Lowry, Jack A. Gilbert, Andrew J. Hoisington

**Affiliations:** 1 Department of Pediatrics and Scripps Institution of Oceanography, University of California San Diego, La Jolla, CA 92037 USA; 2 Department of Integrative Physiology, University of Colorado Boulder, Boulder, CO 80309 USA; 3 Department of Civil, Architectural and Environmental Engineering, University of Texas Austin, Austin, TX 78712 USA; 4 Department of Physical Medicine and Rehabilitation, University of Colorado Anschutz Medical Campus, Aurora, CO 80045 USA; 5 Veterans Health Administration, Rocky Mountain Mental Illness Research Education and Clinical Center (MIRECC), Denver Veterans Affairs Medical Center (VAMC), Denver, CO 80220 USA; 6 Military and Veteran Microbiome Consortium for Research and Education (MVM-CoRE), Denver, CO 80220 USA; 7 School of Medicine, University of Maryland Baltimore, Baltimore, MD 21201 USA; 8 VISN 5 Mental Illness Research Education and Clinical Center (MIRECC), Baltimore, MD 21201 USA; 9 Department of Biology, United States Air Force Academy, Colorado Springs, CO 80840 USA; 10 Departments of Psychiatry and Neurology, University of Colorado Anschutz Medical Campus, Aurora, CO 80045 USA; 11 Center for Neuroscience, University of Colorado Boulder, Boulder, CO 80309 USA; 12 Center for Neuroscience, University of Colorado Anschutz Medical Campus, Aurora, CO 80045 USA; 13 Department of Systems Engineering and Management, Air Force Institute of Technology, Wright-Patterson AFB, OH 45433 USA

**Keywords:** Dormitories, Gut microbiome, Human microbiome, Longitudinal homogenization, Microbiome of the Built Environment, Roommates

## Abstract

**Background:**

The microbiome of the built environment has important implications for human health and wellbeing; however, bidirectional exchange of microbes between occupants and surfaces can be confounded by lifestyle, architecture, and external environmental exposures. Here, we present a longitudinal study of United States Air Force Academy cadets (*n* = 34), which have substantial homogeneity in lifestyle, diet, and age, all factors that influence the human microbiome. We characterized bacterial communities associated with (1) skin and gut samples from roommate pairs, (2) four built environment sample locations inside the pairs’ dormitory rooms, (3) four built environment sample locations within shared spaces in the dormitory, and (4) room-matched outdoor samples from the window ledge of their rooms.

**Results:**

We analyzed 2,170 samples, which generated 21,866 unique amplicon sequence variants. Linear convergence of microbial composition and structure was observed between an occupants’ skin and the dormitory surfaces that were only used by that occupant (i.e., desk). Conversely, bacterial community beta diversity (weighted Unifrac) convergence between the skin of both roommates and the shared dormitory floor between the two cadet’s beds was not seen across the entire study population. The sampling period included two semester breaks in which the occupants vacated their rooms; upon their return, the beta diversity similarity between their skin and the surfaces had significantly decreased compared to before the break (*p* < 0.05). There was no apparent convergence between the gut and building microbiota, with the exception of communal bathroom door-handles, which suggests that neither co-occupancy, diet, or lifestyle homogenization had a significant impact on gut microbiome similarity between these cadets over the observed time frame. As a result, predictive classifier models were able to identify an individual more accurately based on the gut microbiota (74%) compared to skin (51%).

**Conclusions:**

To the best of our knowledge, this is the first study to show an increase in skin microbial similarity of two individuals who start living together for the first time and who are not genetically related or romantically involved. Cohabitation was significantly associated with increased skin microbiota similarity but did not significantly influence the gut microbiota. Following a departure from the occupied space of several weeks, the skin microbiota, but not the gut microbiota, showed a significant reduction in similarity relative to the building. Overall, longitudinal observation of these dynamics enables us to dissect the influence of occupation, diet, and lifestyle factors on occupant and built environment microbial ecology.

**Electronic supplementary material:**

The online version of this article (10.1186/s40168-019-0686-6) contains supplementary material, which is available to authorized users.

## Background

The microbiome of the built environment (MoBE) may have profound impacts on human health and disease both through direct (i.e., exposure to beneficial and pathogenic microorganisms) and indirect mechanisms (i.e., influencing the composition and structure of the human microbiota) [1]. Shared occupancy of an indoor space may result in an increased risk of pathogen exposure, but it could also lead to shared indoor microbial exposures that may shape host immunology. Furthermore, homogenization of lifestyle traits following shared occupancy of an indoor space could influence host-associated microbial similarity of the occupants [2]. Bacterial and fungal communities, characterized using amplicon and metagenomic sequencing approaches in a diverse array of occupied built environments, including homes [3–8], hospitals [9–12], commercial facilities [13, 14], and the International Space Station [15, 16], have demonstrated that building occupants contribute significantly to the indoor microbiome [17, 18]. Built environments are designed for different functions, with non-standard operating conditions that influence both the indoor microbiome as well as occupant health. Seasonal variation in the contribution of outdoor-associated microbes to the indoor microbiome adds further complexity [4, 19, 20], as do differing wind patterns [21–24] and the degree of urbanization [7, 25]. As such, the microbial communities of indoor spaces are diverse and dynamic, which can confound attempts to characterize how shared occupancy of the indoor environment shapes the skin- and stool-associated bacterial communities of human occupants.

The human microbiome is quite individual to each person [26–29] and is rapidly dispersed into the surrounding environment and potentially to other people sharing the same space [30–34]. However, differences in culture, diet, lifestyle, medicine use, geography, and psychological and physical health can influence an individual’s skin and gut microbiota [35–39], potentially confounding our ability to clearly examine how shared occupancy shapes microbial similarity. Schloss et al. [40] found the gut microbiomes in a family of eight shared a core set of operational taxonomic units (OTUs), but, also, each individual contained a set of unique taxa that were distinct enough longitudinally to accurately predict the individual from the group using random forest analysis [37]. For the skin microbiome, Leung et al. [41] observed in cohabitating households that skin microorganisms shared between occupants in the same house ranged from 7–94%. Lax et al. [31] demonstrated that occupants that physically interact with each other share more skin bacterial taxa over time than non-physically interacting occupants that share the same space [28]. In hospitals, skin bacterial taxa associated with the prior occupant of a patient-room were found to be transferred to a new patient when they took up occupancy [9]. The degree of bacterial community similarity between occupants and a building appears to be dependent on both direct human interaction with surfaces and the number of occupants interacting with that surface.

Here, we present a longitudinal study of United States Air Force cadets and their built environment. Our study design was strengthened by sampling a highly homogeneous population that shared many factors such as a standardized diet, lifestyle, housing, and age, which diminished the potential influence of several confounding variables that are known to affect the composition and structure of human microbiota [42–46]. The bacterial community was characterized from both occupants and building surfaces in dyads sharing a room and groups of individuals in different cohabitation locations in a dormitory. The overall goal of this study was to determine how co-occupancy influenced the skin-, gut-, and built environment-associated microbiota of individuals with homogeneous diet, lifestyle, and age. The specific aims of the study were to (1) assess the longitudinal changes to the cadet skin and gut microbiomes, (2) determine how shared occupancy influences the microbiome of the built environment, (3) determine sources of the microbiome of the built environment, and (4) determine accuracy of prediction of occupancy based on comparisons of the skin and gut microbiome of the occupant and the microbiome of the built environment. As the composition and structure of the human microbiome is known to influence health, it is of fundamental importance that we are able to understand how co-occupancy influences the sharing of that microbiota and whether increases in microbial similarity between occupants persists over time.

## Methods

### Cadets’ recruitment

The United States Air Force Academy (USAFA) Institutional Review Board approved the project on 10 May 2016 (FAC20160046H). Cadets were all volunteers, recruited by student peers to be a part of the study. The original study design included four squadrons, each squadron of approximately 100 cadets. Due to the need to meet participant enrollment targets, the study was modified to include four sets of two adjacent squadrons (squadrons 1 and 2, squadrons 3 and 4, squadrons 19 and 20, squadrons 27 and 28). Voluntary surveys were given to the participants at the start of the study and during each week of sampling. The full surveys are included in the supporting information (Additional file 1).

### Sample collection

**Fig. 1:**
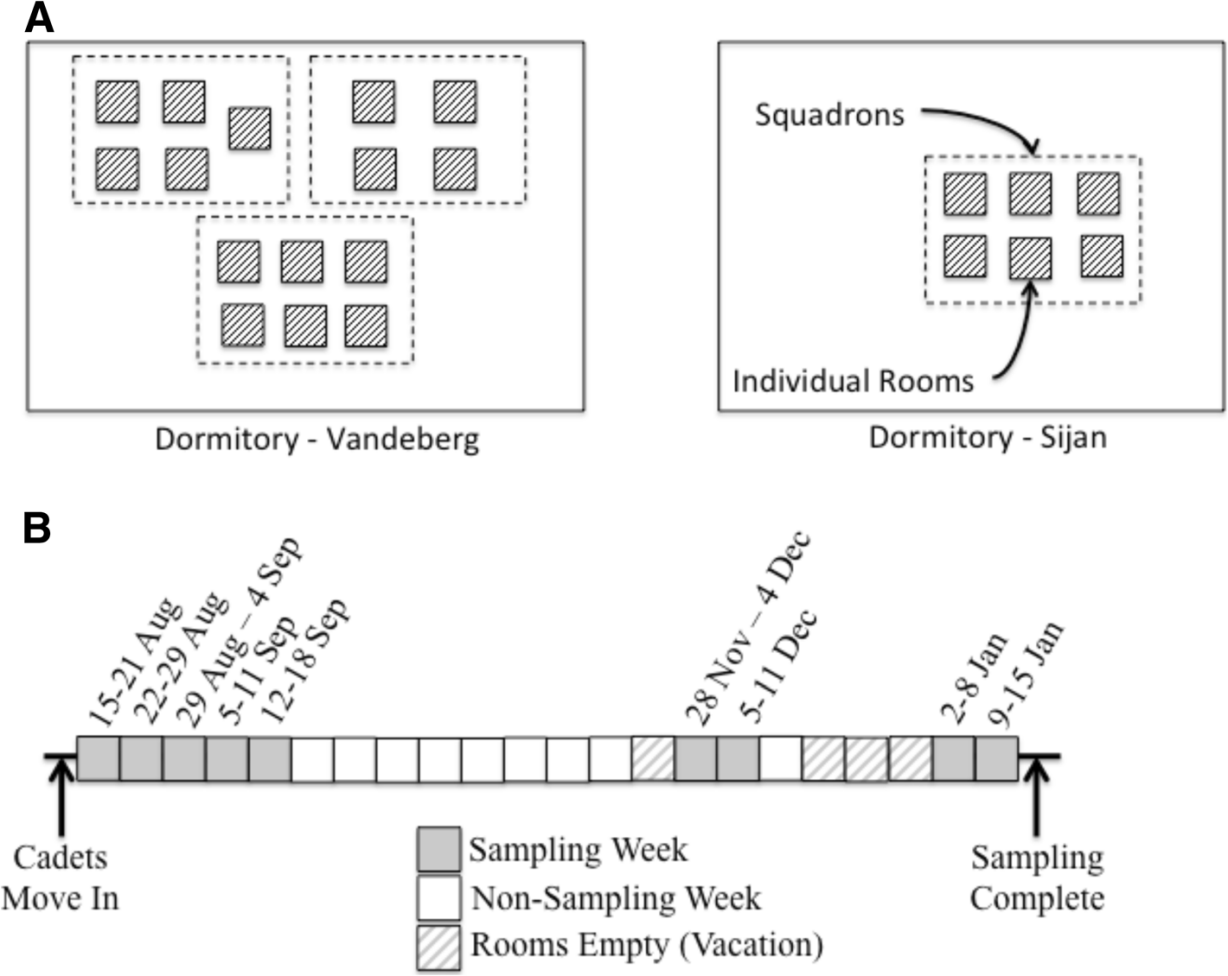
Sampling methodology. **a** Samples were grouped into two dormitories and further defined by squadron and room. **b** Timeline of sampling including the 9 weeks and key events during sampling

Samples for the study were collected weekly at nine different time points: five consecutive weeks at the start of the study, 2 weeks after a November break, and 2 weeks after winter break (Fig. 1, Additional file 2). Human and MoBE samples were collected at the USAFA twice per week and samples from the same week were composited into one sample after sequencing for post-sequencing analysis. A total of 34 cadets occupying 21 rooms participated in this study. Participants were instructed on self-sampling techniques for gut microbiome (a swab of soiled toilet paper) and skin microbiome (a swab of inner elbow) using sterile dual-tipped cotton swabs (Cat. No. 281130, Puritan Medical Products, Guilford, ME, USA). The participant instructions to self-swab are included in the supplemental information (Additional file 3). All self-collected samples were stored in a local freezer for up to 2 days at − 4 °C and then moved to a − 20 °C freezer while awaiting shipment for further processing. Participants were sampled from eight different squadrons (approximately 100 cadets that live and train together) that were located in different locations on campus: squadrons 1 and 2 (adjacent), squadrons 3 and 4 (on the floor below squadrons 1 and 2), squadrons 19 and 20 (in the same building but approximately 400 feet away from squadrons 1–4), and squadrons 27 and 28 in another building (see Fig. 1 and Additional file 2).

Built environment samples were collected from individual rooms with pre-sterilized EnviroMax swabs (Cat. No. 25-88050 PF, Puritan Medical Products) of each participant’s desk, a window sill outside the room (outdoor sample) and a 1-m^2^ vacuum sample (Cat. No. DU-ST-1, Indoor Biotechnologies, Charlottesville, VA, USA) of the dormitory room floor between the desks in each room. In each squadron building, swabs were collected and pooled prior to DNA extraction from two stainless steel bathroom door handles, a floor corner protected from floor cleaning equipment (swabs from a total of four dust samples), a surface above 5 feet that was a surface not normally touched by the cadets (swabs from a total of four dust samples), and the floors of squadron common use areas (thimble vacuum samples; Additional file 2). Six undergraduate research cadets were trained in sampling and performed all room and squadron sampling (see Additional file 2).

### Characterizing features of the built and outdoor environments

Sampling was conducted in two dormitories at the USAFA over a period of 5 months from August 2016 to January 2017; samples were collected during a subset of 9 weeks during this 22-week period (Fig. 1). The heat to each dormitory is supplied through a centrally controlled radiated water system located below the windows in each dormitory room; the rooms are not equipped with air conditioners for cooling. The dormitory rooms were all identical in size (approx. 11 m^2^) and held two participants. Dormitory rooms were cleaned by the occupants as needed. Common areas, to include bathrooms, were cleaned by an external cleaning company each night of the week. One wall in each room contained three windows, two of which were able to be opened by the cadets for natural ventilation.

### Microbiome library preparation

Samples were shipped in coolers with dry ice to the Argonne National Laboratory for DNA processing. The tips of the sampling swabs were broken off into 1.5 ml microtubes containing 500 μl of sterile 1× phosphate-buffered saline (PBS) solution. The swab tips were then immediately vortexed for 10 s. Bacterial DNA was extracted directly from the residual PBS solution using the PowerSoil DNA isolation kit (Mo Bio Laboratories, Carlsbad, CA, USA) following the protocol of Flores et al. [47]. Bacterial DNA from vacuum samples of participants’ rooms and common areas was extracted from dust particles by placing approximately 0.25 g of vacuum filter dust from the thimbles into each well of PowerSoil DNA isolation kits and extracting according to Flores et al. [47]. The V4 region of the 16S rRNA gene (515F-806R) was amplified with region-specific primers that included the Illumina flowcell adapter sequences and a 12-base barcode sequence. Each 25 μl PCR reaction contained the following mixture: 12 μl of MoBio PCR Water (Certified DNA-Free; Mo Bio Laboratories), 10 μl of 5-Prime HotMasterMix (1×), 1 μl of forward primer (5 μM concentration, 200 pM final), 1 μl of Golay Barcode Tagged Reverse Primer (5 μM concentration, 200 pM final), and 1 μl of template DNA [41]. The conditions for PCR were as follows: 94 °C for 3 min to denature the DNA, with 35 cycles at 94 °C for 45 s, 50 °C for 60 s, and 72 °C for 90 s, with a final extension of 10 min at 72 °C to ensure complete amplification. Amplicons were quantified using PicoGreen (Invitrogen, Grand Island, NY, USA) assays and a plate reader, followed by clean-up using UltraClean® PCR Clean-Up Kit (Mo Bio Laboratories) and then quantification using Qubit readings (Invitrogen). The 16S rRNA gene samples were sequenced on an Illumina MiSeq platform (2 × 150 paired-end sequencing, V3 chemistry) at Argonne National Laboratory core sequencing facility according to Earth Microbiome Project (EMP) standard protocols [48]. To verify that no contamination occurred from the DNA extraction kit, 45 PCR-amplified blank controls (i.e., empty extraction wells with only reagents and no input material) were also sequenced along with the other 700 samples in each of the 16S rRNA gene runs. Further, due to the large number of samples, the study sequences were generated on three different sequencing runs. To limit run-to-run influence, the samples were completely randomized by the category (i.e., skin, gut, desk, etc.) of sample and additionally a set of samples (*n* = 18) were sequenced on all three runs. The Shannon alpha diversity values and beta diversity indices (weighted UniFrac) were then compared for the overlapping samples between three runs. We confirmed that the re-run samples within each sample category between the different runs were not significantly different (weighted UniFrac distance ≤ 0.07 in all cases; *p*_permanova_ > 0.05). Sequences and metadata are publically available in the European Bioinfmatics Institute (BioProject ID PRJEB26708) and in QIITA (ID 11740).

### Sequence analysis

For 16S rRNA gene analysis, the 16 million paired-end reads generated for total microbial samples collected (i.e., ~ 5.3 million reads per sequencing run) were joined using join_paired_ends.py script followed by quality-filtering and demultiplexing using split_libraries_fastq.py script in QIIME 1.9.1 [49]. Parameters for quality filtering included 75% consecutive high-quality base calls, a maximum of three low-quality consecutive base calls, zero ambiguous bases, and minimum Phred quality score of 3 as suggested in Bokulich et al. [50]. The final set of demultiplexed sequences was then selected for amplicon sequence variant (ASV) picking using the DeBlur pipeline [51]. In the pipeline, de novo chimeras were analyzed and removed, artifacts (i.e., PhiX) were removed, and ASVs with fewer than 10 reads were removed. Each of the 45 blank controls was assigned very low read counts (< 100 reads/sample) as expected and hence were filtered out of the analyses. The final BIOM file contained 2,170 samples (92% samples retained) of 21,866 unique ASVs with an average of 7,372 reads per sample.

Analysis of the resulting BIOM files was completed in QIIME 1.9.1, R 3.4.2 (phyloseq 1.23.1 and caret 6.0.79 packages), and SourceTracker (in QIIME 1.9.1). For 16S rRNA gene sequences, weighted UniFrac distances [52] were calculated using the ASV count data for the 2,170 samples collected over a period of 5 months from the participants using beta_diversity.py script in QIIME 1.9.1.

In order to understand the convergence patterns between the individual’s microbiome and built environment features in small shared spaces (i.e., 21 rooms), the distributions of weighted UniFrac distance values were plotted as density plots using ggplot2 2.2.1 package (in R) across 9 weeks of sampling. Pairwise comparisons were generated for cadets that shared the same room (roommates) and cadets that do not share the same room (non-roommates). The non-roommates for these comparisons were generated by pairing each cadet with another randomly selected cadet from one of the four squadrons who was not their roommate. Furthermore, gut-gut and skin-skin microbiome convergence patterns were also explored for the cadets who were (1) roommates and (2) non-roommates. Boxplots were generated using geom_box() function in ggplot2 to investigate the association and disassociation patterns between cadets’ microbiome profiles and the built environment microbiomes across shared common spaces of neighboring squadrons. In addition, we compared the gut and skin microbiome profiles of cadets living in neighboring squadrons within the same building (i.e., squadrons 1 and 2, 3 and 4, and 19 and 20) to the built environment samples belonging to squadrons 27 and 28 located in a different building (800 feet away from the first building). The significance of the convergence between sample categories was validated by performing nonparametric analysis of similarity (ANOSIM) [53] using vegan package [54], which generated an *R* statistic and a *p* value, where the *R* value is a statistic for compositional dissimilarity. A lower *R* value indicates higher similarity. For testing the significance of variability patterns of the weighted UniFrac distances (generated between sample categories) over the nine sampled weeks, PERMANOVA was performed in vegan 2.5.1 package of R 3.4.2 [55].

The progressive changes in stability and diversity of microbiomes over the course of sampling within subjects were also evaluated using weighted UniFrac distance matrix in R 3.4.2 [56]. For this, we initially calculated the week-to-week variation (in pairwise manner) using weighted UniFrac distance between same-subject samples in reference to each of the 9 weeks (e.g., week 1 vs week 2, week 1 vs week 3…to…week 1 vs week 9, week 2 vs week 3, week 2 vs week 4…to….week 2 vs week 9). The distances were then plotted as boxplots for each week-wise comparison (paired) using geom_box() function in ggplot2. In reference to each week, the pairwise variations were tested statistically using paired *t* test.

The differentially abundant bacterial ASVs between gut-, skin-, and built environment-associated samples (e.g., desk, dormitory room floor, and outdoor samples) were determined through the analysis of composition of microbiomes (ANCOM) pipeline [57]. Additionally, the number of overlapping ASVs was determined pairwise between different sample categories using subset_samples() and filter_taxa() functions in phyloseq package of *R* by removing all the ASVs not found at least once in both samples [58].

Random forest supervised learning models were used to estimate the predictive power of microbial community profiles to determine participant and room identity using training data from skin, gut, dormitory room floor, desk, and outdoor samples. For each sample type, all the nine time points were aggregated for predicting participant and room identity in order to have enough samples per group to run a significant random forest model. The supervised learning was performed employing two different methodologies, i.e., using cross-validation sample sets in caret package [59] and using out-of-bag (OOB) sample sets in RandomForest package in R [60]. A training set with 70% of the total samples was used for learning models. The feature selection was crosschecked through the recursive feature elimination function in the caret package. Based on each sample category—skin, gut, dormitory room floor, desk, and outdoor—the cross-validation set (30%) was created from the original dataset available for each sample category. Training was accomplished in RandomForest with generation of 1000 trees and prediction accuracy was estimated. Further, in order to supplement the prediction accuracies generated from the validation set, a more robust estimate of generalization error was calculated through OOB error and accuracy (1-OOB) using RandomForest package. The OOB error is an unbiased error rate that predicts the class of a sample using a bootstrap training set without that particular sample. For each training subset used for learning of the models, one third of the samples were left out of the bootstrap sets and hence OOB error was estimated. A lower OOB error indicates a better ability to classify that grouping by microbial community. Finally, RandomForest was used to annotate the top ten most predictive bacterial ASVs for each of the sample categories capable of discriminating between participants and their rooms.

For the SourceTracker models [61], the microbiome profiles of participants’ gut and skin and built environment samples were taken for each room at a given sampling week and consolidated by sample category. The participants’ skin, gut, and outdoor samples were treated as sources for environment sinks that included the desk and dormitory room floor.

## Results

The cohort consisted of USAFA cadets (college students attending a military university), with a certain homogenization of personal characteristics such as lifestyle, diet, and age that are known to influence the human microbiome. Participants did not report any dietary restrictions, had similar sleep patterns per night (mean ± standard deviation (SD); 6.35 h ± 0.86 h), and were 19–21 years old (20.32 ± 0.69), and 92% were male. Diet was not logged in this study but consists of mainly the same family style meals for each participant. Participants were able to select from the food provided during each meal and had limited options for other food during the meals. However, participants can consume other foods, mainly in the form of snacks or on weekends while away from their dormitory rooms. Nearly 25% of the participants were NCAA Division one athletes and all have requirements to maintain physical activity during their time at USAFA, leading to a relatively physically fit cohort. The highly regulated schedule at USAFA requires all cadets to be awake at nearly the same time in the morning.

### Overview of the microbiome of the built environment and its occupants

**Fig 2:**
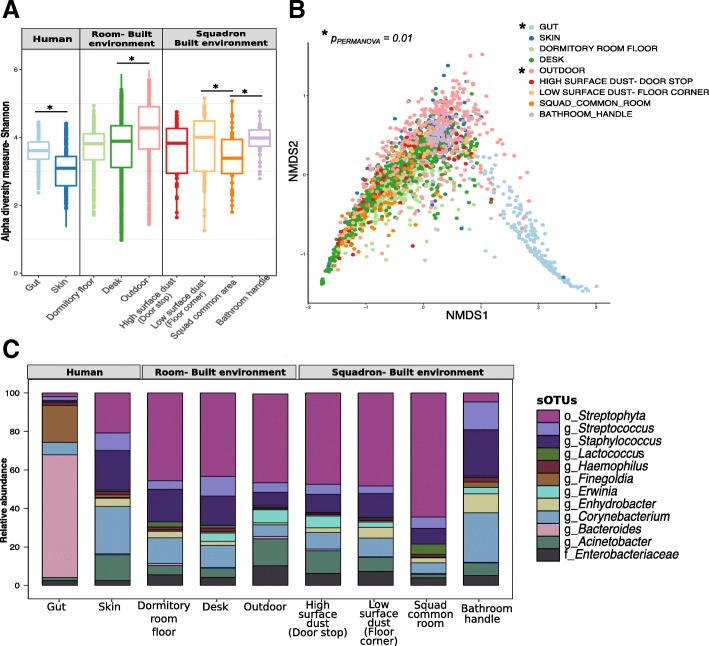
Bacterial diversity analyses using 16S rRNA gene sequences. **a** Shannon alpha diversity within samples by sample category, i.e., human (skin and gut), room-associated built environment samples (desk, outdoor, and dormitory room floor), and squadron-associated built environment samples (bathroom handle, common usage area, high surface dust-door stop, and low surface dust-floor corner) based on the bacterial ASVs. **b** Non-metric multidimensional scaling (NMDS) ordination plot showing variation among sample categories based on the weighted UniFrac distance metric. **c** Distribution of top 20 most abundant ASVs across all the sample categories. Not all ASVs were assigned a genus-level classification; 14 ASVs were assigned to a genus (“g”), 4 were assigned to an order (“o”), and 2 were assigned to a family (“f”)

Human and environmental sampling from 9 weeks between August 2016 and January 2017 provided 2,170 samples for analysis. Samples were human skin, human gut, dormitory room desk, dormitory room floor, dormitory hallway doorstop, dormitory hallway floor corner, dormitory common area, dormitory bathroom handle, and an outdoor window lintel. Alpha diversity differed significantly between human and built environment (BE) sample types (*p*_anosim_ = 0.001, Shannon), whereby the BE samples were more diverse, followed by gut, and then skin (Fig. 2a, Additional file 4). Alpha diversity was significantly similar within each sample type over time (*p*_PERMANOVA_ *≥* 0.08).

The microbial community beta diversity of the single outdoor location (window lintel) was significantly different from the indoor surfaces (*p*_permanova_ = 0.01*,* weighted UniFrac), while BE surfaces within a cadet dormitory (i.e., desks and floors) were not significantly different (*p*_permanova_ > 0.05*,* weighted UniFrac). The 20 most abundant ASVs were significantly differentially abundant across the sample categories (*p* < 0.05; Fig. 2c). Gut-associated bacterial communities, the only anaerobic sampling location in the present study [62], formed a distinct cluster (*p*_permanova_ *= 0.01,* NMDS ordination) compared to the skin and BE samples (Fig. 2b). Skin and BE samples were enriched in *Streptococcus* and *Staphylococcus* (Fig. 2c), which is consistent with previous observations [8, 32, 63, 64]. *Propionibacteriaceae* were present in low relative abundance in skin samples, unlike other skin-associated studies [65–67], which is most likely due to primer bias associated with the V4 region of the 16S rRNA gene [68].

Additionally, we identified differentially abundant ASVs (*p*_BH-FDR Corrected_ < 0.05) between nine sample types, at each of the nine time points. We identified a consistent bacterial signature associated with each sample type over all the time points. For instance, *Corynebacterium* was enriched on both skin and bathroom handle over time compared to other sample types, *Bacteroides* was at a significantly greater proportion in the gut, *Propionibacterium* was more abundant on the skin and bathroom handles, and ASVs belonging to order *Rickettsiales* and *Streptophyta* were enriched in the outdoor samples (Additional file 5). We also identified ASVs that were unique to particular sample types, especially outdoor samples, and only with specific time points. ASVs from genera *Modestobacter* (1.5%) and *Cloacibacterium* (1.1%) were significantly enriched in outdoor and floor corner samples only at week 1 (Additional file 5). An ASV belonging to family *Acetobacteraceae* (7.2%) was also significantly enriched in outdoor samples at week 1; *Flavisolibacter* (0.4%) was enriched in outdoor samples in week 2; *Micrococcaceae* (0.4%) was enriched in the dormitory room floor samples in week 3; ASVs from *Deinococcus* (2.4%) and *Methylobacterium* (4.5%) were significantly enriched in outdoor samples in week 4; an ASV from family *Aeromonadaceae* (14.7%) was significantly enriched in outdoor samples at week 7. Genus *Oscillospira* (0.74%) was found to be associated with gut at weeks 8 and 9 (Additional file 5).

**Fig. 3:**
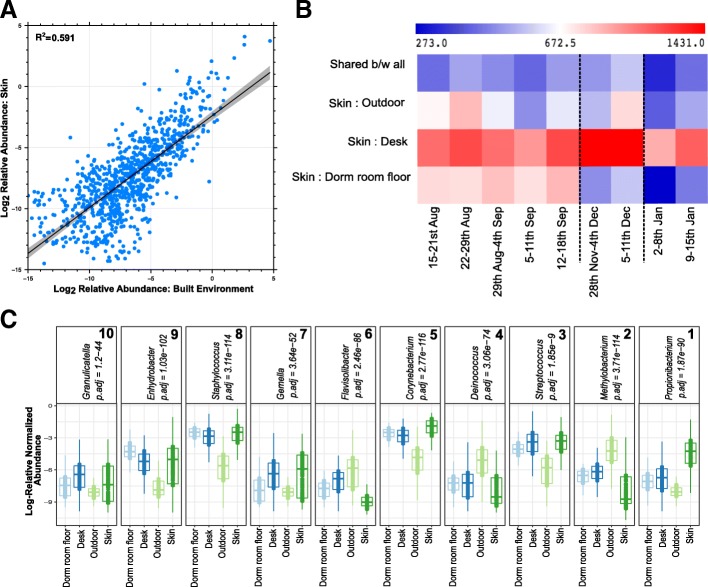
Distinctive bacteria relative abundances across sample category and week. **a** Plot of log_2_-transformed average relative abundances in the cadets’ skin and built environment samples for all ASVs. **b** Shared ASVs heatmap for skin and individual built environment samples, i.e., desk, dormitory room floor, and outdoor across the temporal sampling series. Total samples week 1 = 1107, week 2 = 1207, week 3 = 1102, week 4 = 982, week 5 = 1211, week 6 = 1431, week 7 = 1429, week 8 = 914, week 9 = 1149. **c** Differentially abundant genera between skin and built environment samples as identified by ANCOM, which are then ranked from 1 to 10 (right to left) based on feature importance score based on random forest models

Overall, the bacterial signatures differentiating the sample type categories, i.e., dormitory room floor, desk, gut, skin, and outdoor, were consistent in both the roommates (*n* = 1504; all sample types) and non-roommate datasets (*n* = 1016; all sample types) (Additional file 6). Across the entire study, the relative abundance of ASVs between the skin and built surfaces showed an *R*^*2*^ correlation of 0.59 (log_2_ of relative abundance; Fig. 3a). The skin samples shared maximum numbers of ASVs with desk with no significant decrease after the first break (10 days between week 5 to week 6; *p* = 0.1); however, the number of shared ASVs was reduced after the second break (22 days between week 7 to week 8; *p* = 0.03) (Fig. 3b). The sharing between skin and dormitory room floor demonstrated a significant reduction (*p_BH-FDR Corrected_ < 0.05) following both breaks (Fig. 3b). A multigroup ANCOM revealed ten bacterial genera that were significantly differentially abundant across the skin, desk, dormitory room floor, and outdoor samples (Fig. 3c). *Propionibacterium*, *Corynebacterium*, *Streptococcus*, and *Staphylococcus* were significantly more abundant in the skin samples; *Deinococcus*, *Methylobacterium*, and *Flavosolibacter* were significantly more abundant in the outdoor samples, while the dormitory room floor and desk samples were mostly enriched for *Corynebacterium*, *Staphylococcus*, *Enhydrobacter*, and *Gemella* (Fig. 3c). Gut samples, when compared to both skin and built environment samples, contained higher abundance of anaerobic genera including *Bacteroides*, *Blautia*, *Coprococcus*, and *Ruminococcus* (Additional file 6). Meanwhile, the skin and built environment samples were significantly enriched with *Corynebacterium* in addition to *Staphylococcus* and *Streptococcus*.

### Longitudinal changes to cadet skin and gut microbiome

**Fig. 4:**
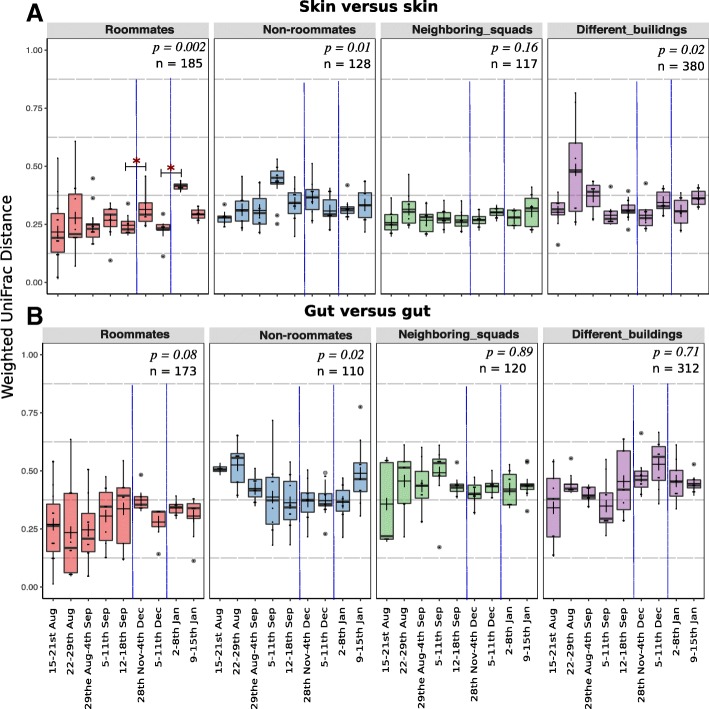
Boxplots showing distribution of weighted UniFrac distances calculated between roommates versus between non-roommates collected over 9 data points across a period of 5 months. **a** Skin-to-skin and **b** gut-to-gut comparison between two individuals sharing the same room (roommates), individuals not having roommate association (non-roommates, i.e., randomly generated dataset in which each cadet was paired with a cadet who was not their roommate), individuals in the neighboring squadrons (i.e., squadron pairs in the same building, i.e., 1 and 2, 3 and 4, 19 and 20), and individuals residing in squadrons in different buildings (i.e., above squadron pairs compared with squadrons 27 and 28 located in a different building, which is 400 feet away). PERMANOVA *p* values (*p*_permanova_) are mentioned for longitudinal comparisons of weighted UniFrac distances (skin versus skin or gut versus gut). Blue dashed lines represent the two vacations, which break the continuous sampling points. Two asterisks over two time points (i.e., after the vacation) indicate that the difference between the UniFrac distance measures at those specific time points is significant (*p* < 0.05) based on the PERMANOVA test. The dark lines inside the boxes of boxplots are medians and “+” represents the mean

Previous studies have reported similarity in skin microbial community structure in cohabitating family members [31, 41, 69] and cohabitating partners [70, 71]. The skin microbiota from cohabitating roommates was significantly more similar (ANOSIM *R* = 0.231, *p*_anosim_ < 0.05) compared to non-roommates (ANOSIM *R* = 0.474, *p*_anosim_ < 0.01, Fig. 4a). As observed in a previous longitudinal cohabitation study [41], the amount of similarity was non-standard across the study. In that study, Leung et al.[41] hypothesized the difference in similarity between cohabitating members might depend on personal factors. The present study supports that similarity of skin microbiomes between cohabitating individuals and herein expands those results to non-related individuals. Specifically for this study, the connection between roommates began as we started sampling and the level of connections between the roommates may have gotten stronger or weaker depending upon occupant behavior or other personal factors. Future longitudinal studies could investigate the differences in the similarity of skin microbiomes between cohabitating individuals in more depth and could register the time individuals spend together and the proximity between the occupants.

The convergence patterns of the skin microbiome between the two roommates were significantly affected by the mandatory evacuation of the dormitories during the Thanksgiving and Winter Holiday breaks (*p*_permanova_ = 0.002, Fig. 4a). Immediately after the two breaks when the cadets did not cohabitate (18 November 2016 to 28 November 2016; 11 December 2016 to 2 January 2017), the similarity between the skin microbiota of roommates was significantly reduced compared to before the breaks (ANOSIM *R* = 0.569, *p*_anosim_ < 0.05 after the first break, and ANOSIM *R* = 0.512, *p*_anosim_ < 0.05 after the second break). Notably, after the second break, which was over twice as long as the Thanksgiving break, the roommates’ skin microbial communities were the most dissimilar of the entire study (*p*_permanova_ < 0.05; Fig. 4b). Likewise, using within-cadet pairwise weighted UniFrac comparisons across the weeks, the skin microbiota from week 1 were most dissimilar when compared to the weeks immediately following the breaks (*p*_*t* test_ < 0.05, Additional file 7). The reduction in skin microbial community similarity after the break was limited to roommates (Fig. 4b). Cadets who did not share a living space (randomized pairwise comparison of non-roommates excluding the designated roommate pairs) had no increased skin microbial similarity over time and no associated reductions in similarity across the two breaks (Fig. 4a).

The gut microbiota of roommates was not affected by the two break periods and also remained stable longitudinally (*p*_permanova_ *= 0.08*, Fig. 4b). Non-roommate gut microbiota were significantly different over the study (*p*_permanova_ *= 0.02,* Fig. 4b*)*, with an apparent reduction in microbiome dissimilarity until the last week of the study*.* The individual pairwise comparisons of weighted UniFrac distances within the gut microbiota from week 1 to the weeks after their breaks were significant after both breaks (*p*_*t* test_ < 0.05, Additional file 7).

### Shared occupancy influences the BE microbiota

A qualitative overview of skin, gut, built environment, and outdoor sample beta diversity values longitudinally using NMDS ordination based on the weighted UniFrac metric revealed a distinct cluster of gut samples across all nine sampled weeks (*p*_permanova_ < 0.05) (Additional file 8). The skin and built environment samples did not significantly separate (*p*_permanova_ > 0.05; except for the outdoor samples), suggesting that the built environment microbiota likely originate predominantly from the skin (Additional file 8). Ordination of environmental samples from squadron buildings revealed a tight clustering for floor corner, squadron common area, and door stop samples. The bathroom handle samples ordinated as a separate group (*p*_permanova_ < 0.05) until the November sampling, after which there was a visible blending (*p*_permanova_ > 0.05) of bathroom handle samples with other environment samples (Additional file 8).

**Fig. 5:**
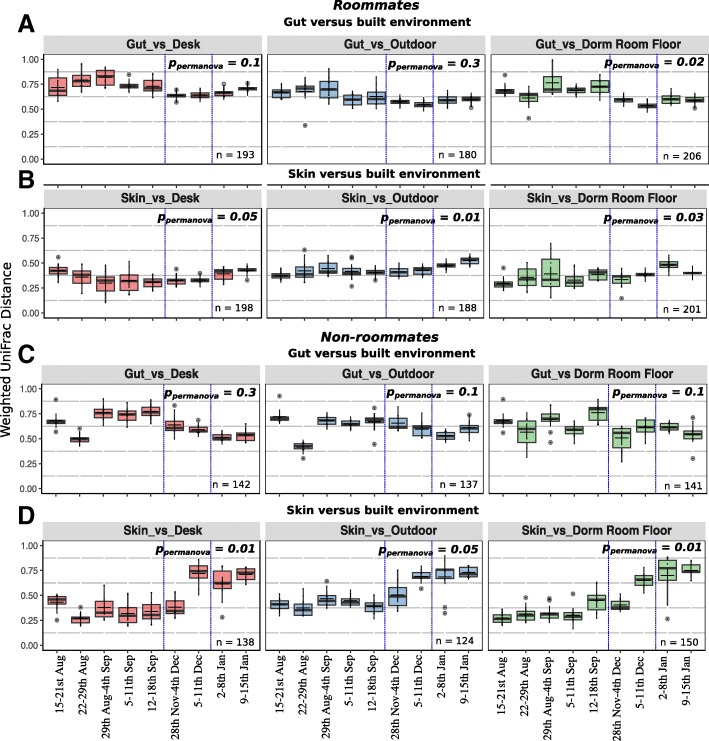
Boxplots showing distribution of weighted UniFrac distances calculated between human and built environment samples collected over 9 data points across a period of 5 months. Distribution of weighted UniFrac distances between **a** gut (both roommates) and built environment samples associated with the dorm room (desk, dormitory room floor, outdoor), **b** skin (both roommates) and dorm room samples, **c** gut (non-roommates) and dorm room samples, and **d** skin (non-roommates) and dorm room samples. Weighted UniFrac distances were calculated from the dataset of 1,515 roommate samples and 1,263 non-roommate samples. *n* values in each panel indicate the total number of pairs used for different sample categories in weighted UniFrac distance calculations. PERMANOVA *p* values (*p*_permanova_) are labeled for the comparison of weighted UniFrac distances (for each pair, i.e., human vs built environment) between the 9 weeks of sampling. Blue dashed lines represent the two vacation breaks during which the cadets vacated the rooms. The dark lines inside boxplots are the medians and “+” represents the mean, which in most cases overlapped with the medians

The microbial communities found in the dormitory rooms (i.e., roommates) were more similar to the skin (ANOSIM, *R* = 0.312 for skin versus desk, *R* = 0.406 for skin versus dormitory room floor, and *R* = 0.514 for skin versus outdoor) than the gut microbiota of the occupants (ANOSIM, *R* = 0.583 for gut versus desk, *R* = 0.612 for gut versus dormitory room floor, and *R* = 0.552 for gut versus outdoor) (Fig. 5a, b, Additional file 9). The higher similarity between skin and BE compared to gut and BE was interestingly evident across the non-roommate dataset as well (Fig. 5c, d, Additional file 9). The skin and surface microbiota were relatively more similar to the occupants of a room relative to non-roommates (Fig. 5b, d, Additional file 9). The weighted UniFrac distances between gut and the BE for all of the nine weeks of sampling did not significantly change for roommates for the desk (*p*_permanova_ = 0.1) or outdoor (*p*_permanova_ = 0.3) (Fig. 5a, Additional file 9), which suggests no significant distribution of gut bacteria to these surfaces.

**Fig. 6:**
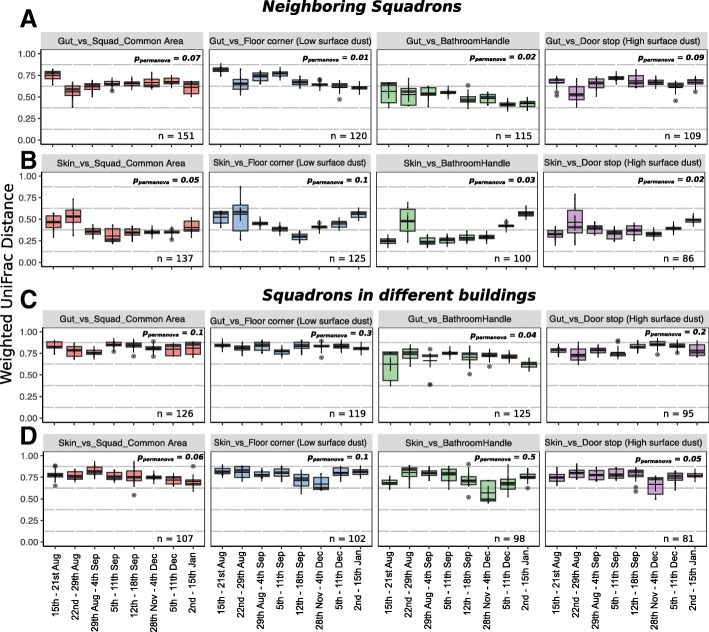
Boxplots showing distribution of weighted UniFrac distances calculated between human and built environment samples collected over 8 data points across a period of 5 months in publicly shared spaces, i.e., squadrons. Distribution of weighted UniFrac distances between **a** gut and built environment samples, i.e., squadron common area, floor corner (low surface dust sample), bathroom handle, and door stop (high surface dust sample) for neighboring squadrons, **b** skin and built environment samples for neighboring squadrons, **c** gut and built environment for squadrons located in different buildings, and **d** skin and built environment for squadrons located in different buildings. PERMANOVA *p* values (*p*_permanova_) are labeled for the comparison of weighted UniFrac distances (for each pair, i.e., human vs built environment) among the eight weeks sampled. The dark lines inside the boxes of boxplots are medians and “+” represents the mean, which in most cases overlapped with the medians. The neighboring squadrons are the ones within the same building and with adjacent hallways, i.e., squadron pairs 1 and 2, 3 and 4, 19 and 20. The comparisons for squadrons in different buildings are between the gut and skin microbiome profiles of cadets living in the neighboring squadrons (abovementioned pairs) to the built environment samples belonging to squadrons 27 and 28 located in a different building (400 feet away from the first building)

Additionally, the microbiome was analyzed to determine the similarity between the gut or skin microbiota of cadets, and that of squadron-shared built environment samples, which included a bathroom handle, door stop (high, surface dust sample), floor corner (low, surface dust sample), and the common area vacuum sample (Fig. 6). After quality filtering, there were not enough samples to provide adequate statistical analysis for the last 2 weeks; hence, we aggregated samples from the 2 to 8 January and 9 to 15 January into a single time point, i.e., 2 to 15 January (Fig. 6). We compared the convergence patterns between the microbial profiles of cadets and the BE across shared common spaces of squadron pairs who had neighboring hallways and resided in the same building (i.e., squadrons 1 and 2, 3 and 4, 19 and 20; all plotted together). The gut samples showed significant microbial community convergence over time with both the bathroom handle (*p*_permanova_ = 0.02) and floor corner (*p*_permanova_ = 0.01) only in squadrons located in the same building (i.e., squadrons 1 and 2, 3 and 4, 19 and 20; Fig. 6a). Gut samples had a greater similarity to the bacterial profile on the bathroom handle (ANOSIM *R* = 0.392, *p*_anosim_ < 0.05) compared to the floor corner samples (ANOSIM *R* = 0.512, *p*_anosim_ < 0.001) (Fig. 6a, c). The gut microbiota displayed significant variation with the bathroom handle microbiota over the duration of the study for both neighboring squadrons (*p*_permanova_ = 0.02; Fig. 6a) and squadrons in different buildings (*p*_permanova_ = 0.04; Fig. 6c) while the comparison of the skin microbiota with the bathroom handle microbiota did not follow this trend (Fig. 6b, d). However, relative to the gut, the skin microbiota were more similar to the bathroom handle at all the time points (weighted UniFrac, ANOSIM *R* = 0.254, *p*_anosim_ < 0.05) (Fig. 6a, b).

The cadets’ skin microbiota was significantly similar to the samples in the squadron common area, for the squadrons in the same building (ANOSIM *R* = 0.289, *p*_anosim_ < 0.05) (Fig. 6b). The squadron common area is a space used for meetings and leisure activities for the cadets in the same squadron. In addition, we compared the gut and skin microbial profiles of cadets living in the neighboring squadrons (abovementioned pairs) to the BE samples belonging to squadrons 27 and 28 located in a different building (400 feet away from the first building). The skin microbiome compared to common rooms in different buildings did not show the same level of microbiome similarity (ANOSIM *R* = 0.601, *p*_anosim_ *>* 0.05*)* (Fig. 6d). Indeed, no significant temporal convergence was observed between any cadet’s gut and skin microbiota (from squadrons 1 and 2, 3 and 4, 19 and 20) and the surfaces in a different squadron building (27 and 28) that they did not inhabit (Fig. 6c, d).

### Sources of the microbiome of the built environment

**Fig. 7:**
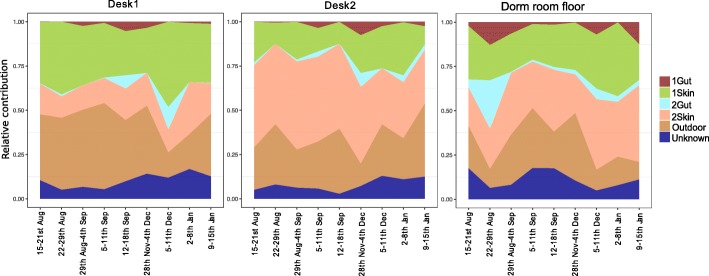
Sourcetracker analysis shows the sources of bacteria found on built surfaces. The surfaces include the dormitory room floor and occupant-specific desks (i.e., desk1, desk2). The four sources include the two occupants’ skin and gut samples and the outdoor surface (which is representative of external environment microbiota)

Each room was comprised of two desks (approximately 2 m apart), where each desk belonged to one occupant. Sourcetracker analysis revealed the occupant’s skin microbiota was a major source of ASVs to a cadet’s own desk (37.8 ± 0.02%, Fig. 7), while their roommate contributed significantly less (17.0 ± 0.01%, Fig. 7). In the long-term sampling, skin microbiota from both occupants contributed a similar percentage to the composition of dust samples from the dorm room floor (28.5 ± 0.02%, i.e., sum total for both occupants, Fig. 7).

The outdoor microbiota contained a significantly greater percentage of ASVs with an unknown source (55.2 ± 0.03%). Longitudinally, the outdoor microbiota contribution was greater early in the summer for all BE sinks, which may have been due to an increase in open windows in the dormitories as a result of an increase in warm weather; however, the spike of outdoor bacteria during January might have been from cadets opening their windows to lower the indoor temperature or simply due to the reduction in occupant-supplied indoor microbes during the break.

### Prediction of occupants

We applied a random forest classifier to determine whether the microbial community can predict an individual or a specific room. Gut samples were 74% accurate in identifying an individual from which they originated (OOB_gut_ = 0.26, 74% probability of classifying a sample correctly when the sample was not used in training the model), which contrasts with the skin microbiota (OOB_skin_ = 0.49, 51%, Additional file 10). The desk-associated bacterial community predicted the correct occupant with 71% accuracy (OOB_desk_ = 0.29). These results suggest that the desk maintains a microbial signature that is more consistent over time compared to the skin [63, 64, 72].

Additionally, we tested the diagnostic capacity of the dormitory room floor and outdoor sample for predicting the room from which they originated. As expected, the dormitory room floor microbiota were able to predict the room of origin with an accuracy of 81% (OOB_common_room_ = 0.19) (Additional file 10). Vacuuming the dust that collects on the dormitory room floor provides a sample of a long-term microbial signature and may be a better sampling approach for the BE than surface swabs for prediction of long-term occupancy [8]. The outdoor samples, as expected, had a lower prediction accuracy of the room’s identity (OOB_outdoor_ = 0.58, 42%) (Additional file 10). For the gut-based RandomForest model (trained to predict an individual’s identity), the top ten discriminative features were assigned to the genera *Prevotella*, *Parabacteroides*, *Oscillospira*, *Bacteroides* (*caccae*), *Dialister*, and *Butryicimonas*. The predictive model for the participant’s identity using skin microbiome data included discriminative ASVs associated with *Corynebacterium*, *Propionibacterium*, *Micrococcus*, *Actinomyces*, *Aeromondaceae*, and *Acetobacteraceae*. Similarly, a desk-based training model for predicting rooms included discriminative ASVs assigned to *Corynebacterium*, *Acinetobacter*, *Anerococcus*, *Coprococcus*, *Rothia*, and *Lactobacillus*. The discriminative ASVs for the model predicting room based on dormitory room floor data included genera *Pseudomonas*, *Macrococcus*, *Jeotgalicoccus*, *Corynebacterium*, and *Aerococcaceae*. Overall, built environment-based RandomForest models for desk and dormitory room floor shared discriminative features with skin, which again indicated the connection between skin and those built environment microbiomes.

## Discussion

This longitudinal study enabled a detailed exploration of the influence of lifestyle, diet, and architectural homogenization of the microbial sharing between individuals and with the BE. Within each sample type, the alpha and beta diversity remained quite stable over time. While roommates did not display a significant increase in the similarity of either the gut or skin microbiota over time, they were significantly more similar than non-roommates. The desk-associated microbiota was significantly more similar to the occupant that used that desk compared to any other cadet, while the shared floor space between the beds was more similar to both roommates than to any other cadet. In a longitudinal study of the built environment prior to and post-opening of a hospital, an increase in alpha diversity was observed in samples taken from locations with human skin contact [9]. In the present study, the cadet rooms were previously occupied and therefore the surfaces likely held residual microbial biomass originating from prior occupants.

Similar to the skin, the gut microbiota of all of the roommates did not converge across the study. We have no rational explanation for the observed congruity in the gut microbiota observed by a subset of cadets as they shared no specific traits that would suggest similarity. The gut microbiome did not appear to be a substantial source of bacteria to most BE surfaces, with the exception of bathroom handles. The result here of transfer of gut microbiome to the restroom door handle was not observed in the Flores et al. [73] restroom study, though that study did not have a longitudinal design. It is possible the transfer of the gut microbiome is a slow process that can be detected only after multiple weeks. Despite the decreased dissimilarity between the gut microbiome and the restroom door handle, the dominant microbiome on the restroom door handle was still the skin microbiome as observed by Flores et al. [73]. It is unlikely the skin microbiome was directly transferred from the antecubital fossa to the door handles, but instead consisted of microorganisms from that hand that are shared with the sampling site in the present study.

Gut-associated microbiota were enriched in *Bacteroides* (70% of the top 20 ASVs), which is consistent with other Western adult microbiome studies [42, 44], and may be suggestive of a Westernized animal-based diet [45]. All of the cadets that responded to the initial survey (74% response rate) indicated they were not vegetarian or vegan (*n* = 25). In summary, cohabitation and the homogenization of lifestyle, activity, and diet were not major drivers of gut microbiome dynamics.

There were two breaks (vacations) during the semester when the cadets were required to vacate their rooms. These breaks enabled observation of temporal microbial stability following the absence of the occupants. Indeed, the absence and its duration were both associated with significant shifts in the human microbiota, but also in the similarity between the skin and BE surface microbiota, which had significantly declined immediately after each vacation. This is likely due to either the acquisition of new skin-associated bacteria during the break, a reduction in bacterial sharing between occupants or reduced exposure to the lifestyle, diet, and activity homogenization while at the academy [74]. While it is potentially more likely that a reduction in sharing and homogenization could have influenced the similarity, it is also possible that the skin microbiota could have been altered by the environments that cadets interacted with during the vacation, as geography can influence the human microbiota [14, 23, 75]. However, the gut microbiota were not influenced by the vacations, which suggests remarkable longitudinal stability in the face of a substantial reduction in diet and activity homogeneity (especially during Thanksgiving and Winter Holiday, which usually are associated with substantial food consumption). Previous studies have also reported highly stable gut microbiota over time [76].

The human-associated bacterial profile was highly predictive of the individual, with gut microbiome more predictive than the more variable skin microbiota. Within a dormitory room, the desk microbiota was able to predict the cadet that most regularly interacted with it almost as well as the cadets’ gut microbiota predicted them; meanwhile, the floor between the cadets’ beds could predict the two cadets that lived in that room with over 80% accuracy. The desks were swabbed in entirety once per week, providing a composite temporal sample, while the floor sample comprised vacuumed dust. Interestingly, the floor and the desk were not new when the cadets moved in, and so, may have contained bacteria from the prior occupant, as has been seen in hospital rooms [9].

The relative abundance of bacterial ASVs was significantly correlated between the skin and the BE samples, and those surfaces with which an individual cadet interacted shared a more personalized subset of the skin bacteria of that cadet. However, skin samples were swabbed from the antecubital fossa (inner elbow), whereby desquamation is the most likely cause of microbial dissemination, as opposed to direct physical interaction with a BE surface. An alternative, already mentioned above in the new text, is that there are shared microorganisms in the antecubital fossa and skin surfaces that touch the desk. Overall, the gut microbiota was significantly more similar to the bathroom door handle, which might suggest direct contact with the hands of the cadets following their use of the bathroom. Yet, the bathroom handle was sampled on the exterior of the door, likely contacted prior to using the bathroom indicating persistent gut microorganisms on the hands or a lack of cleaning over time. The door handle being on the exterior was presumably not heavily influenced by resuspension of gut microorganisms that may have settled in the bathroom. As in many college dormitories, cadets at USAFA are free to use any bathroom and each floor has several available to use; as such, there is no way to identify those that deposited these samples on the door handles. Overall, the microbiota in common rooms used for training and social activities were more similar to the skin microbiota of cadets that lived in that building than those occupying the other building.

Limitations of the study include a large sampling effort of over 5,000 samples that required multiple sequencing runs. Previously, others have noted a run-to-run variation in sequencing [14], which was not observed in this study based on the analysis conducted. The present study also was limited by one skin site that is not directly in contact with the built environment. The antecubital fossa was chosen due to its relatively stable microbiome over time, in comparison to the highly variable hand microbiome [77]. Sampling the microbiome of other skin sites might have resulted in different findings. Finally, the study design required gathering informed consent when the cadets returned from summer break and moved into their new rooms with different roommates. It took several days to consent all of the participants, and therefore, the study did not have an initial baseline before roommates started to live together. To alleviate that known issues, the study did sample over 100 locations in the built environment at USAFA prior to the cadets’ occupancy. Unfortunately, an error in shipping results in a loss of all of those samples.

Strengths of this study include homogeneity of the sampling population in terms of lifestyle, diet, activity levels, age, physical condition, and occupation. Although those measures were not completely uniform, this study does represent a unique cohort that limited bias in the microbiome compared to other human microbiome efforts. Additionally, the extraction processes, primers, and sequencing technology were chosen to maximize the ability to conduct comparisons between this study and other research in the field. Likewise, human and built environment sampling locations were selected based on previous research conducted multiple laboratories to again allow comparisons between studies. Finally, the study included temporal changes over a 6-month period in multiple built environment and human microbiome sampling sites which enabled some determination of microbiome stability and increased the ability to investigate causes of longitudinal microbiome perturbations.

## Conclusions

This 5-month longitudinal microbial analysis of USAFA cadets and their BE indicates a significant microbial dispersion from the host to the BE. The degree of interaction an individual has with a particular surface will significantly increase their microbial sharing with that surface. Despite no clear temporal convergence, cohabitating roommates had greater skin-associated microbial community similarity when compared to non-cohabiting individuals in the same building. While the gut microbiota is quite stable over time, perturbation in diet and lifestyle associated with vacations had a significant impact on the skin microbiota. Overall, the gut microbial profile was more predictive of a person’s identity than the skin microbiota; also, the desk and floor were predictive of which cadets lived in that room. Human Microbiome-Wide Associations Studies [78] use statistical approaches to identify microbial taxa or functions that are associated with disease or health. Identifying such organisms in the BE will require much more refined assessments of the health of occupants, which was not attempted in this study. Future work will attempt to determine if the microbial sharing observed between occupants, and with the BE, has any impact on the health or behavioral characteristics of the cadets. If so, then it is possible that the microbial traits of the environment could be manipulated to augment health outcomes [2, 39, 79] with skin microbiome as a preliminary target for researchers in the short term.

## Additional files

Additional file 1: Initial and weekly participant surveys. (DOCX 809 kb)

Additional file 2: Locations of room and squadron sampling in this study. (DOCX 4991 kb)

Additional file 3: Sampling protocols for skin and gut swab microbial sampling. (DOCX 169 kb)

Additional file 4: Distribution of Shannon and Simpson alpha diversity indices across different sample categories collected from rooms over a period of nine sampled weeks. (DOCX 2787 kb)

Additional file 5: Relative abundance of significantly different ASVs between the nine sample types at each time point, identified using ANCOM analyses. (DOCX 683 kb)

Additional file 6: Distinctive bacteria relative abundances across sample categories as generated by analysis of composition of microbiomes (ANCOM). (DOCX 587 kb)

Additional file 7: Weekly instability (i.e., week-to-week variation within subjects) based on the weighted UniFrac distance matrix. (DOCX 307 kb)

Additional file 8: A) Non-metric multidimensional scaling (NMDS) plot based on weighted-UniFrac metrics pattern for the different sample categories (i.e., gut, skin, desk, dormitory room floor and outdoor) collected from the rooms over a period of 5 months. B) Non-metric multidimensional scaling (NMDS) plot based on weighted UniFrac metrics pattern for the different sample categories (i.e., bathroom door handle, high surface dust-door stop, low surface dust-floor corner, and squad common area) collected from common areas over a period of 5 months. (DOCX 3097 kb)

Additional file 9: Density plots comparing the distributions of weighted UniFrac distance measures calculated for 9 data points between A,B) skin and built environment and C,D) gut and built environment in roommate and non-roommate datasets. (DOCX 862 kb)

Additional file 10: Confusion matrices for random forest models generated based on different sample categories. (DOCX 354 kb)

## Abbreviations

ANCOM: Analysis of composition of microbiomes
ANOSIM: Analysis of similarity
ASVs: Amplicon sequence variants
BH-FDR: Benjamini-Hochberg false discovery rate
HMP: Human Microbiome Project
MIRECC: Rocky Mountain Mental Illness Research Education and Clinical Center
MoBE: Microbiome of the built environment
MVM-CoRE: Military and Veteran Microbiome Consortium for Research and Education
NMDS: Non-metric multidimensional scaling
OOB: Out-of-bag error
PBS: Phosphate-buffered saline
PCR: Polymerase chain reaction
PERMANOVA: Permutational multivariate analysis of variance
QIIME: Quantitative Insights Into Microbial Ecology
SD: Standard deviation
USAFA: United States Air Force Academy
