# Supplementary material for: Longitudinal homogenization of the microbiome between both occupants and the built environment in a cohort of United States Air Force Cadets
Source: Microbiome. 2019 May 2;7:70. doi: 10.1186/s40168-019-0686-6 (PMC6498636; doi:10.1186/s40168-019-0686-6)
Supplement: Supplementary file 1 — Initial and weekly participant surveys. (DOCX 809 kb) [file 40168_2019_686_MOESM1_ESM.docx]

**Figure 1. Initial Participant Survey.** Provided to Cadets during the first week of the study.

**Figure 2. Weekly Participant Survey.** Provided to Cadets during each week of sampling.
