# Supplementary material for: Longitudinal homogenization of the microbiome between both occupants and the built environment in a cohort of United States Air Force Cadets
Source: Microbiome. 2019 May 2;7:70. doi: 10.1186/s40168-019-0686-6 (PMC6498636; doi:10.1186/s40168-019-0686-6)
Supplement: Supplementary file 2 — Locations of room and squadron sampling in this study. (DOCX 4991 kb) [file 40168_2019_686_MOESM2_ESM.docx]

**Figure 1. Location of microbiome sampling for participant rooms and squadron areas.**
