# Supplementary material for: Longitudinal homogenization of the microbiome between both occupants and the built environment in a cohort of United States Air Force Cadets
Source: Microbiome. 2019 May 2;7:70. doi: 10.1186/s40168-019-0686-6 (PMC6498636; doi:10.1186/s40168-019-0686-6)
Supplement: Supplementary file 3 — Sampling protocols for skin and gut swab microbial sampling. (DOCX 169 kb) [file 40168_2019_686_MOESM3_ESM.docx]

**Figure 1. Skin Swab Protocol.** The exact instructions provided to Cadet participants, given as a hard copy with each of the weekly sampling swabs.

**Figure 2. Gut Swab Protocol.** The exact instructions provided to Cadet participants, given as a hard copy with each of the weekly sampling swabs.
