# Supplementary material for: Longitudinal homogenization of the microbiome between both occupants and the built environment in a cohort of United States Air Force Cadets
Source: Microbiome. 2019 May 2;7:70. doi: 10.1186/s40168-019-0686-6 (PMC6498636; doi:10.1186/s40168-019-0686-6)
Supplement: Supplementary file 4 — Distribution of Shannon and Simpson alpha diversity indices across different sample categories collected from rooms over a period of nine sampled weeks. (DOCX 2787 kb) [file 40168_2019_686_MOESM4_ESM.docx]

******

**Figure 1. Distribution of Shannon and Simpson alpha diversity indices across different sample types, i.e. skin, gut, outdoor, desk, dormitory room floor, collected from rooms over a period of nine sampled weeks.**
