# Supplementary material for: Longitudinal homogenization of the microbiome between both occupants and the built environment in a cohort of United States Air Force Cadets
Source: Microbiome. 2019 May 2;7:70. doi: 10.1186/s40168-019-0686-6 (PMC6498636; doi:10.1186/s40168-019-0686-6)
Supplement: Supplementary file 5 — Relative abundance of significantly different ASVs between the nine sample types at each time point, identified using ANCOM analyses. (DOCX 683 kb) [file 40168_2019_686_MOESM5_ESM.docx]

**Additional file 5:** Relative abundance of significantly different ASVs between the nine samples types at each time point, identified using ANCOM analyses. Please, note that no differentially abundant ASVs were identified at week 6 and due to low number of samples in week 8 and 9, both were aggregated for ANCOM analyses.
