# Supplementary material for: Longitudinal homogenization of the microbiome between both occupants and the built environment in a cohort of United States Air Force Cadets
Source: Microbiome. 2019 May 2;7:70. doi: 10.1186/s40168-019-0686-6 (PMC6498636; doi:10.1186/s40168-019-0686-6)
Supplement: Supplementary file 6 — Distinctive bacteria relative abundances across sample categories as generated by analysis of composition of microbiomes (ANCOM). (DOCX 587 kb) [file 40168_2019_686_MOESM6_ESM.docx]

**Figure 1: *Distinctive bacteria relative abundances across sample types.*** *Distribution of significantly (BH-FDR corrected, p < 0.05) different bacterial genera across 5 sample types, i.e. dormitory room floor, desk, gut, outdoor, and skin, between (A) roommates and (B) non-roommates.*
