# Supplementary material for: Longitudinal homogenization of the microbiome between both occupants and the built environment in a cohort of United States Air Force Cadets
Source: Microbiome. 2019 May 2;7:70. doi: 10.1186/s40168-019-0686-6 (PMC6498636; doi:10.1186/s40168-019-0686-6)
Supplement: Supplementary file 7 — Weekly instability (i.e., week-to-week variation within subjects) based on the weighted UniFrac distance matrix. (DOCX 307 kb) [file 40168_2019_686_MOESM7_ESM.docx]

***Figure 1:*** *Weekly instability (week-to-week variation within subjects). Weighted-UniFrac distance between same-subject samples taken 1 sampling point apart (in pairwise manner) is plotted against these sampling week pairs. The dark line inside the boxes of boxplots are medians and “+” stands for mean, which in most cases are overlapping in this figure. * represents statistically significant variation (p < 0.05) for the pairwise comparison.*
