# Supplementary material for: Longitudinal homogenization of the microbiome between both occupants and the built environment in a cohort of United States Air Force Cadets
Source: Microbiome. 2019 May 2;7:70. doi: 10.1186/s40168-019-0686-6 (PMC6498636; doi:10.1186/s40168-019-0686-6)
Supplement: Supplementary file 8 — A) Non-metric multidimensional scaling (NMDS) plot based on weighted-UniFrac metrics pattern for the different sample categories (i.e., gut, skin, desk, dormitory room floor and outdoor) collected from the rooms over a period of 5 months. B) Non-metric multidimensional scaling (NMDS) plot based on weighted UniFrac metrics pattern for the different sample categories (i.e., bathroom door handle, high surface dust-door stop, low surface dust-floor corner, and squad common area) collected from common areas over a period of 5 months. (DOCX 3097 kb) [file 40168_2019_686_MOESM8_ESM.docx]

**Figure 1. Non-metric multidimensional scaling (NMDS) plot based on weighted-UniFrac metric.** *(A) The clustering pattern for the different sample types i.e. gut, skin, desk, dormitory room floor and outdoor collected from the rooms over a period of over 5 months revealed distinct clustering for gut samples and an overlapping clustering pattern for skin- and built environment-associated samples. (B) Among the samples associated with larger shared spaces of squadrons, bathroom handle samples formed a separate cluster which merged with door stop, floor corner, and squadron common area samples over time.*
