# Supplementary material for: Longitudinal homogenization of the microbiome between both occupants and the built environment in a cohort of United States Air Force Cadets
Source: Microbiome. 2019 May 2;7:70. doi: 10.1186/s40168-019-0686-6 (PMC6498636; doi:10.1186/s40168-019-0686-6)
Supplement: Supplementary file 9 — Density plots comparing the distributions of weighted UniFrac distance measures calculated for 9 data points between A,B) skin and built environment and C,D) gut and built environment in roommate and non-roommate datasets. (DOCX 862 kb) [file 40168_2019_686_MOESM9_ESM.docx]

**Figure 1.** *Density plots comparing the distributions of weighted UniFrac distance measures calculated for 9 data points between (A) skin and built environment of shared occupants, i.e. roommates, (B) skin and built environment of individuals not having roommate association, i.e. non-roommates, (C) gut and built environment of roommates and (D) gut and built environment of non-roommates. PERMANOVA tests of variance generated significant p values, i.e. < 0.05 for all comparisons over time except for gut comparisons to the built environment for roommates at 10,000 permutations of the dataset of 1,515 samples and 1,263 samples for roommates and non-roommates, respectively. PERMANOVA p values (p_permanova_) are labeled for the comparison of weighted UniFrac distances (for each pair, i.e., human vs built environment) between nine sampling weeks. n values in each panel state the total number of pairs used for different sample types in weighted UniFrac distance calculations.*
