# Supplementary material for: Longitudinal homogenization of the microbiome between both occupants and the built environment in a cohort of United States Air Force Cadets
Source: Microbiome. 2019 May 2;7:70. doi: 10.1186/s40168-019-0686-6 (PMC6498636; doi:10.1186/s40168-019-0686-6)
Supplement: Supplementary file 10 — Confusion matrices for random forest models generated based on different sample categories. (DOCX 354 kb) [file 40168_2019_686_MOESM10_ESM.docx]

***Gut bacterial microbiota can predict the individual’s identity better than skin***

Additionally, the RandomForest models were used to calculate discriminative ASVs leading to differentiation between different participants as well as different rooms. For the gut-based RandomForest model (trained to predict an individual’s identity), the top ten discriminative features were assigned to the genera *Prevotella, Parabacteroides, Oscillospira, Bacteroides (caccae), Dialister,* and *Butryicimonas*. The predictive model for the participant’s identity using skin microbiome data included discriminative ASVs associated with *Corynebacterium*, *Propionibacterium, Micrococcus, Actinomyces*, *Aeromondaceae* and *Acetobacteraceae*. Similarly, a desk-based training model for predicting rooms included discriminative ASVs assigned to *Corynebacterium, Acinetobacter, Anerococcus, Coprococcus, Rothia,* and *Lactobacillus*. The discriminative ASVs for the model predicting room based on dormitory room floor data included genera *Pseudomonas, Macrococcus, Jeotgalicoccus, Corynebacterium,* and *Aerococcaceae*. Overall, built environment-based RandomForest models for desk and dormitory room floor shared discriminative features with skin, which again indicated the connection between skin and those built environment microbiomes.

**Figure 1. Confusion matrix to validate the prediction accuracies of random forest models using the 16S rRNA gene data.** *Heatmaps representing the confusion matrices generated (A) from each training dataset i.e. gut, skin and desk for predicting participant’s identity and (B) from built environment training datasets* (*i.e. dormitory room floor, desk and outdoor*) *used for prediction of the room’s identity. The title for each plot includes % accuracy followed by OOB error. The confusion matrix is a way of tabulating the number of misclassifications i.e., the number of predicted classes that ended up in a wrong classification bin based on the true classes. The diagonal elements show the number of correct classifications for each class. The off-diagonal elements provide the misclassifications.*
